# Timing of antiretroviral therapy and regimen for HIV-infected patients with tuberculosis: the effect of revised HIV guidelines in Malawi

**DOI:** 10.1186/1471-2458-14-183

**Published:** 2014-02-20

**Authors:** Hannock Tweya, Anne Ben-Smith, Mike Kalulu, Andreas Jahn, Wingston Ng’ambi, Elizabeth Mkandawire, Layout Gabriel, Sam Phiri

**Affiliations:** 1 The International Union Against Tuberculosis and Lung Disease, Paris, France; 2 The Lighthouse Trust, P.O. Box 106, Lilongwe, Malawi; 3 Institute of Social and Preventive Medicine, University of Bern, Bern, Switzerland; 4 Department of Biomedical Informatics, University of Pittsburg, P.O. Box 31563, Lilongwe, Malawi; 5 Central Monitoring and Evaluation Division/Department for HIV and AIDS, Ministry of Health, Lilongwe, Malawi; 6 International Training and Education Center for Health (I-TECH), Lilongwe, Malawi; 7 University of Washington, Seattle, USA

**Keywords:** TB/HIV co-infected, Antiretroviral therapy, ART/PMTCT guidelines

## Abstract

**Background:**

In July 2011, the Malawi national HIV program implemented the integrated antiretroviral therapy (ART) and prevention of mother-to-child transmission (PMTCT) guidelines. Among the principle goals of the guidelines were increasing ART uptake among TB/HIV co-infected patients and treating TB/HIV patients with a different drug regimen. We, therefore, assessed the effects of the new guidelines on ART uptake, the factors associated with ART uptake and the frequency of ARV-related adverse events in TB/HIV co-infected patients.

**Methods:**

This was an observational cohort study using routine program data. All ART-naïve adult TB/HIV co-infected patients starting TB treatment over the six months preceding and following implementation of 2011 integrated ART/PMTCT guidelines were included.

**Results:**

A total of 685 adult TB/HIV co-infected patients were registered in the study; 377 (55%) before and 308 (45%) after the implementation of the new guidelines. ART uptake increased from 70% (240/308) before implementation of the new guidelines to 78% (262/377) after the inception of the new guidelines (*P*=0.013). The proportion of TB patients initiating ART within two weeks of starting TB treatment increased from 30% before implementation of the new guidelines to 46% after implementation of the new guidelines (p <0.001). The median time from the start of TB treatment to ART initiation dropped from 16 days (IQR 14-31) before the new guidelines to 14 days (IQR 9-20; p = 0.004) after implementing the new guidelines. Factors associated with ART uptake were enrolment in HIV care before starting TB treatment and being a retreatment TB patient. The overall frequency of ARV-related adverse events was higher in patients on d4T/3TC/NVP (35%) than those on TDF/3TC/EFV (25%) but not significantly different (*P*=0.052).

**Conclusion:**

Implementation of the 2011 Malawi Integrated ART/PMTCT guidelines was associated with an overall increase in ART uptake among TB/HIV patients and with an increase in the number of patients initiating ART within two weeks of starting their TB treatment. However, the reduction in time between initiating TB treatment and starting ART was small suggesting that further measures must be implemented to facilitate ART uptake. Early enrolment in HIV care provides opportunities for timely ART initiation among TB patients.

## Background

Human immunodeficiency virus (HIV) and tuberculosis (TB) are the two of the major epidemics in sub-Saharan Africa. In 2010, the TB/HIV co-infection rate in TB patients was about 44% in the African region and as high as 82% in high TB/HIV burden countries [1]. The TB case-fatality rate is between 16% and 35% in TB/HIV co-infected individuals not receiving antiretroviral therapy (ART) in Africa [2].

Previous studies showed reductions in HIV-associated mortality and morbidity among TB/HIV co-infected individuals who start ART early [3,4]. Recent clinical trials demonstrated that early initiation of ART was safe, and was associated with increased survival [5-7]. In 2010, the World Health Organisation (WHO) revised guidelines reflecting this growing body of evidence [8]. These guidelines recommend initiation of ART among TB/HIV co-infected patients, irrespective of CD4 cell count, within eight weeks of starting TB treatment.

Malawi, a poor landlocked country in Africa, is severely affected by the dual epidemic of HIV and TB. In 2012, the estimated incidence of TB was 163 persons per 100,000 population [9] and HIV prevalence among TB patients was 66%, resulting in a considerable proportion of early deaths in individuals on ART attributed to undiagnosed TB [10]. With this high TB burden, the performance of the national TB programme is negatively impacted.

The use of ART results in reduction in rates of TB in HIV cohorts [11,12]. Malawi embarked on rapid scale up of ART in 2004 and, by 2012, almost 390,000 individuals were alive and on ART. Despite such remarkable progress in the provision of ART, only 46% of TB/HIV patients started ART while on TB treatment in 2010 [13], mainly due to the limited integration of TB and HIV services. In July 2011, the Malawi national HIV program adopted the 2010 WHO guidelines and implemented treatment guidelines (Malawi Integrated Guidelines for Clinical Management of HIV, 2011, First Edition [14]) to increase access to quality ART and PMTCT services. For the treatment of TB/HIV infection, the guidelines recommend the initiation of ART within two weeks of the start of TB treatment and the provision of the antiretroviral drugs (ARVs) Tenofovir, Lamividine and Efavirez (TDF/3TC/EFV) to ART-naïve patients. Also, the guidelines recommend both the provision of Isoniazid preventive therapy (IPT) for pre-ART patients to reduce the incidence of TB, and intensified TB case finding (ICF) for all patients in pre-ART and ART follow-up to enable early diagnosis and treatment of TB. Since its implementation, it is unclear whether the guidelines have led to an increase in the overall proportion of TB/HIV patients starting ART during TB treatment and within two weeks of initiation of TB treatment, and, ultimately, improved TB treatment outcomes. In addition, there is limited information on the comparative effects of the ARV regimens given to TB/HIV patients based on the Malawi national ART/PMTCT guidelines. We, therefore, assessed the effects of the national guidelines on ART uptake in TB patients, and the factors associated with ART uptake at the largest TB registration centre in Malawi. We documented the ARV regimen that TB patients received and compared the frequency of ARV-related adverse events between TB/HIV co-infected individuals initiating a Stavudine, Lamivudine and Nevirapine (D4T/3TC/NVP) ARV drug regimen to those initiating a TDF/3TC/EFV regimen.

## Methods

### Study design

We conducted an observational cohort study using routine program data. All ART-naïve adult TB/HIV co-infected patients who started TB treatment and were managed at Martin Preuss Centre (MPC) over the six months preceding and following implementation of 2011 integrated Malawi national ART/PMTCT guidelines were eligible for inclusion in the study. The study period extended from January 2011 to December 2011.

### Setting

The study was conducted at MPC, the largest TB registration site in the country. MPC is an integrated TB/HIV clinic located in Malawi’s capital city, Lilongwe. The clinic, described in detail previously [15], functions in partnership with the Lilongwe District Hospital and has three units: HIV testing and counselling, ART, and TB; the latter unit includes sputum submission. Almost 66% of the TB patients in Lilongwe are registered at MPC. Approximately 3,200 TB patients are registered annually at the clinic. HIV ascertainment among TB patients is 95%. Diagnosis of TB is based on clinical examination, sputum smear microscopy, chest radiography and other investigations as appropriate for extra-pulmonary disease. Once diagnosed with TB, patients are recorded in the national TB register by the district TB officer. TB treatment is initiated at MPC but patients can choose to complete their treatment at one of the 18 peripheral health facilities in Lilongwe District.

TB/HIV co-infected patients are started on ART regardless of CD4 cell count. Before implementation of the new ART/PMTCT guidelines in July 2011, TB/HIV co-infected patients were initiated on a regimen of d4T/3TC/NVP; from July 2011, patients were initiated on TDF/3TC/EFV. TB/HIV co-infected patients are seen monthly for clinical assessment and collection of their ARVs and TB drugs. At each clinic visit, patients are screened for common symptoms and ARV-related adverse events using a symptom/drug side-effect checklist comprising of anaemia, jaundice, lactic acidosis, hepatitis, peripheral neuropathy, diarrhoea, abdominal pain and skin rash. TB treatment outcomes (cured, treatment complete, transferred-out, lost to follow-up, treatment failure and died) are updated in the TB paper registers. TB and ART services are completely integrated; HIV-positive and HIV-negative TB patients are managed by the same clinical officers.

### Data collection and statistical analysis

Data were extracted from the clinic’s electronic data system and from TB registers and analysed in Stata 12.0. Descriptive analysis was used to explore the proportion of TB/HIV co-infected patients starting ART and developing ARV-related adverse events. In the time-to-event analysis, patient were entered in the analysis at the time of TB registration and observation time ended either at the time of ART initiation, death, lost to follow-up, transfer to other TB facility or completion of TB treatment, whichever came first. We estimated the cumulative probability of starting ART while accounting for competing risk of death. Competing risks proportional hazards regression models were used to estimate the adjusted associations of patient characteristics and implementation of new guidelines and time to ART initiation. For the purpose of this study, we considered only adverse events registered as resulting from ARVs according to the clinical officer’s or physician’s clinical judgment. For the analyses, the first occurrence of each adverse event was considered the end point of the outcome. We compared the frequency of ARV-related adverse events between TB/HIV co-infected individuals initiating a D4T/3TC/NVP drug regimen and those initiating a TDF/3TC/EFV drug regimen using the chi-square test. A level of significance of P ≤ 0.05 was used.

### Ethical considerations

The study was approved by the Malawi National Health Science Research Committee in Lilongwe, Malawi, and The Union Ethic Advisory Board in Paris, France. As the study used routine programme data, both ethics boards provided a waiver of individual informed consent. Data for the study did not include any personal identifiers.

## Results

### Study population

A total of 1,069 adult TB/HIV co-infected patients were registered for TB treatment during the study period, of which 384 (36%) were excluded as they were already on ART. Of the remaining 685 (64%) study participants, 377 (55%) started TB treatment before the implementation of the new ART/PMTCT national guidelines and 308 (45%) started after implementation of the guidelines. There were no significant differences in sex, median age at TB registration, TB classification and TB category in the study participants who started TB treatment before and after implementation of the new ART guidelines (Table 1).

**Table 1:** Characteristics of HIV-infected individuals with tuberculosis registered at MPC before and after implementation of the 2011 Malawi national ART/PMTCT guidelines, January 2011 and December 2011

	**Before (n = 377)**		**After (n = 308)**		**P-value**
Female (%)	132	35%	106	34%	0.870
Age (years) Median (IQR)	34	(29–40)	34	(29–39)	0.459
15-34	204	54%	157	51%	0.413
35+	173	46%	151	49%	
Type of TB^₣^					
Extrapulmonary	88	25%	65	22%	0.545
Smear-positive pulmonary	104	29%	83	28%	
Smear-positive pulmonary	164	46%	149	50%	
TB category^β^					
New	341	91%	282	92%	0.646
Retreatment	33	9%	24	8%	
HIV test^£^					
Before TB diagnosis	208	60%	167	56%	0.312
After TB diagnosis	141	40%	133	44%	
Enrolled in HIV care					
Before TB diagnosis	53	14%	59	18%	0.084
After TB diagnosis	336	86%	262	82%	
Started ART					
By 2 weeks	112	30%	142	46%	<0.001
By 6 month	257	68%	237	77%	0.013
Total	262	70%	240	78%	0.013

^₣^32 had missing data on TB types, ^β^5 had missing data TB category, ^£^36 had unknown time of HIV test.

### ART uptake before and after implementation of the new guidelines

**Figure 1:**
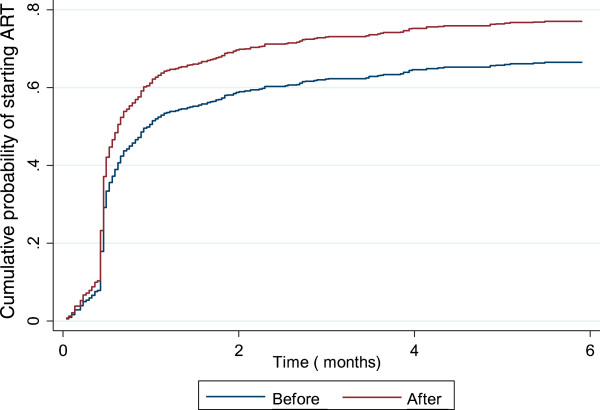
Time from start of TB treatment to initiation of ART among HIV patients receiving TB treatment at Martin Preuss Centre Clinic, pre- and post-implementation of the 2011 Malawi ART/PMTCT guidelines.

The proportion of patients starting ART while on TB treatment increased from 70% (262/377) before implementation of the new guidelines to 78% (240/308) after implementation (p = 0.013) (Table 1). After implementation of the new guidelines, by two weeks after the start of their TB treatment, 46% of TB patients had started ART, compared to 30% before implementation (p <0.001). A total of 115 TB patients registered before implementation of the new guidelines were not started on ART; of these, 97 (84%) did not start ART although they were in care throughout their TB treatment, 13 (11%) died, 4 (3%) transferred to other TB facilities and 1 (<1%) was lost to follow-up. After implementation of the new guidelines, 68 of the newly registered TB patients did not start ART; of these, 61 (90%) were in care throughout their TB treatment, 3 (4%) died, 3 (4%) transferred to other TB facilities and 1 (1%) was lost to follow-up. The median time to ART initiation after the start of TB treatment dropped from 16 days (IQR 14–31) before implementation of the new guidelines to 14 days (IQR 9–20; p = 0.004) after implementation. Figure 1 shows the cumulative probability of starting ART up to 6 months after initiating TB treatment, accounting for competing risks. The probability of starting ART at 2 weeks and 2 months after initiating TB treatment increased from 32% and 58% to 40% and 64%, respectively, after implementation of the new guidelines.

After controlling for gender, age, TB classification, time of enrolment in HIV care and TB patient category, patients starting TB treatment after the implementation of the 2011 national ART/PMTCT guidelines were 1.38 (95% CI 1.15-1.65) times more likely to start ART compared to TB/HIV patients registered before implementation of the new guidelines (Table 2). Patients in HIV care at the start of their TB treatment were more likely to be initiated on ART than patients not in HIV care at the start of their TB treatment (adjusted subhazard ratio 1.72; 95% CI 1.36-2.18).

**Table 2:** Competing risk regression modelling of time from TB initiation to starting ART in TB patients co-infected with HIV at MPC, January 2011 and December 2011

**Characteristics**	**N (%)**	**SHR**^**€ **^**(95% CI)**		**Adjusted SHR**^**€ **^**(95% CI)**	
Guideline change					
Before	377 (55%)	1	(ref)	1	(ref)
After	308 (45%)	1.34	(1.13 – 1.61)	1.38	(1.15–1.65)
Sex					
Male	447 65%)	1	(ref)	1	(ref)
Female	238 (35%)	0.87	(0.72 – 1.04)	0.87	(0.71–1.06)
Age (years)	685	0.99	(0.98 – 1.01)	0.99	(0.98–1.00)
Type of TB^₣^					
Extrapulmonary	153 (23%)	1.15	(0.92 – 1.43)	1.16	(0.92–1.46)
Smear–positive pulmonary	187 (29%)	1.04	(0.84 – 1.29)	1.04	(0.84–1.30)
Smear–positive pulmonary	313 (48%)	1	(ref)	1	(ref)
TB patient category					
New	623 (92%)	1	(ref)	1	(ref)
Retreatment	57 (8%)	0.70	(0.52 – 0.95)	0.66	(0.49–0.91)
Enrolled in HIV care					
Before TB diagnosis	107 (16%)	1.77	(1.43 – 2.20)	1.72	(1.36–2.18)
After TB diagnosis	578 (84%)	1	(ref)	1	(ref)

SHR^€^ = SubHazard ratios ^₣^45 PTB had unknown smear results; 5 cases had missing data for TB patient category.

### ARV regimens and ARV-related adverse events

A total of 502 patients started ART during their TB treatment. All patients starting ART before the implementation of the 2011 national ART/PMTCT guidelines received the D4T/3TC/NVP drug regimen. After implementation of the guidelines, 174 (61%) of TB/HIV patients received the TDF/3TC/EFV drug regimen and 110 (39%) of patients received the D4T/3TC/NVP drug regimen. ARV drug regimen was not recorded in 4 patients registered prior to implementation of the new guidelines.

We explored the ARV-related adverse events recorded during the ART follow-up visits among the 284 TB/HIV patients who started ART after implementation of the national guidelines. The median follow-up time was 7.9 months (IQR 4.3-7.8); 40 patients were lost to follow-up, 5 stopped ART, 49 transferred out to other TB facilities and 19 died. A total of 88 ARV-related adverse events were observed in 82 (29%) patients (Table 3). The overall frequency of ARV-related adverse events was higher in patients on the d4T/3TC/NVP regimen (35%) than those on the TDF/3TC/EFV regimen (25%), but not significantly different (P = 0.052). Peripheral neuropathy was more frequent among patients on the D4T/3TC/NVP drug regimen than among those on the TDF/3TC/EFV regimen (22% vs 9%). Vomiting was more common among patients on the D4T/3TC/NVP regimen than among patients on the TDF/3TC/EFV regimen (10% vs 4%). We also noted a case of hepatitis and one case of jaundice in patients on the TDF/3TC/EFV regimen. The ARV drug regimen was changed in 3 (2%) and 8 (10%) patients on the TDF/3TC/EFV regimen and the D4T/3TC/NVP regimen, respectively.

**Table 3:** **Distribution of ARV-related adverse events by ARV drug regimen among TB patients receiving ART at MPC, Malawi**^**£**^

**ARV-related adverse effects**	**Total (n = 284)**		**d4T/3TC/NVP (n = 110)**		**TDF/3TC/EFV (n = 174)**	
Total with one or more ARV-related adverse events^*^	82	29%	39	35%	43	25%
Anorexia	6	2%	2	2%	4	2%
Leg pain/numbness	40	14%	24	22%	16	9%
Jaundice	1	0%	0	0%	1	1%
Skin rash	9	3%	5	5%	4	2%
Vomit	22	8%	4	4%	18	10%
Hepatitis	1	0%	0	0%	1	1%
Abdominal pain	5	2%	2	2%	3	2%
Fever	5	2%	2	2%	3	2%
Other	9	3%	5	5%	4	2%

*P-value = 0.052; ^£^ARV = antiretroviral.

## Discussion

This is the first formal evaluation of the effect of the 2011 Malawi national ART/PMTCT guidelines on ART uptake, factors associated with ART uptake and use of the TDF/3TC/EFV regimen in TB/HIV patients. Implementation of the new guidelines was associated with an increase in ART uptake in TB patients. However, the reduction in time to starting ART was marginal. The prevalence of ARV-related toxicities was higher in patients receiving the D4T/3TC/NVP drug regimen than those receiving the TDF/3TC/EFV regimen and more drug changes occurred in patients on the former regimen. These findings support the feasibility and safety of initiating ART early in TB/HIV co-infected patients and the use of the recommended TDF/3TC/EFV regimen in real-life clinical settings.

After implementation of the 2011 national integrated ART/PMTCT guidelines, there was an increase (from 30% to 46%) in the proportion of patients initiating ART within two weeks of the start of their TB treatment and an increase in the overall ART uptake among TB patients from 70% to 78%. We believe that the increased ART uptake and reduced time to ART initiation was due to the implementation of these new guidelines. During the study period, there were no major concurrent interventions in TB patients apart from the implementation of the guidelines. Although the overall national burden of TB patients co-infected with HIV has been decreasing over the years, the proportion of TB patients co-infected with HIV remained constant at the study site (65%) during the study period. Furthermore, both ascertainment of HIV and enrolment in HIV care prior to TB registration were similar before and after implementation of the guidelines. Even before the implementation of the new guidelines, MPC, unlike most TB/ART clinics in Malawi, provided ART to patients two weeks after starting TB treatment [15], which meant that TB patients at MPC already had a shorter than average waiting period to initiate ART. Therefore, although we observed a significant increase in ART uptake in TB patients at MPC after implementation of the new guidelines, the effect of the guidelines might have been obscured. Nevertheless, the national recommendation probably encouraged more rapid initiation of ART in TB patients among whom there may be a high mortality rate.

Similar to other studies [16,17], even after the implementation of the new guidelines, a sizeable proportion (22%) of TB/HIV co-infected patients did not start ART, suggesting the need for clinic-based interventions. Among those who did not initiate ART, 90% did not start ART in spite of being in care throughout their TB treatment. Also, the reduction in the time between TB registration and initiation of ART start was marginal and may not necessarily have had clinical benefits. The findings clearly indicate that the tracking of patients through the continuum of care must be improved. Intensified educational efforts aimed at reminding the TB clinic staff and patients about the importance of ART and addressing stigma and fear, and also better clinic patient flow are needed to improve ART uptake. Even as the uptake of ART among TB/HIV co-infected is scaled up, much morbidity and mortality in TB/HIV patients could be prevented by applying the “Three I’s”: intensified case finding, infection control, and isoniazid preventive therapy. In Malawi’s national HIV programme, pre-ART and ART patients are screened for TB during their follow-up. By June 2013, 94% of all patients retained on ART had been screened for TB at their last clinic visit and 58% of all patients retained in pre-ART care had received IPT.

Our results also showed an independent association between being in HIV care and starting ART. TB patients who were in HIV care prior to TB registration were more likely to start ART compared to those who enrolled in HIV care after TB registration. One possible explanation is those patients who were already in HIV care might have been easier to track through the continuum of care and to get started on ART. Thus, increasing early HIV testing and enrolment in HIV care would likely provide opportunities for timely ART initiation and prevention of TB disease. Retreatment TB patients were less likely to start ART, possibly because of the model of TB/ART service delivery at the clinic. All retreatment TB patients are admitted in a TB ward for two months. This TB ward is separate from the MPC TB outpatient clinic, and ART services are not provided on the ward. Thus, these patients are only offered ART after their two months of intensive TB treatment. Provision of ART services to TB patients admitted on the TB ward would not only facilitate ART uptake among this category of patients but also improve their TB treatment outcomes.

Almost 39% of TB/HIV co-infected patients were initiated on the D4T/3TC/NVP regimen after implementation of the revised Malawi national ART/PMTCT guidelines. The new guidelines recommend the D4T/3TC/NVP regimen for ART-naïve adult TB/HIV co-infected patients with anaemia (with haemoglobin levels of < 8 g/dl); however, none of the TB patients in this study who started on the D4T/3TC/NVP regimen had documented anaemia. Co-administration of the D4T/3TC/NVP regimen and the rifampicin-containing anti-tuberculosis therapy containing may lead to drug interactions. Previous studies showed a reduction in the levels of NVP in the presence of rifampicin [18], possible loss of virologic capabilities and increased drug resistance [19,20]. It is therefore important for all ART providers to follow the new national treatment guidelines and ensure that all TB/HIV co-infected patients are started on the recommended TDF/3TC/EFV regimen.

Almost a quarter of the patients in this study developed ARV-related adverse events during the follow-up period. The prevalence of adverse events was higher in patients on the D4T/3TC/NVP regimen than in patients on the TDF/3TC/EFV regimen and more patients on the D4T/3TC/NVP regimen were switched to the other ARV regimens, suggesting the comparative safety of the TDF/3TC/EFV regimen in this population. Peripheral neuropathy and vomiting were frequently reported adverse events. Neuropathy is usually caused by D4T and may be more likely when D4T and isoniazid are co-administered [21]. With the observed high prevalence of neuropathy, it is likely that the neuropathy was induced by co-administering of D4T and isoniazid. Due to short and long term toxicity of D4T, the Malawi integrated ART/PMTCT guidelines specifically recommends TDF. Considering the high prevalence of neuropathy, the D4T containing regimen should only be given when no alternative options are available. Due to high cost, provision of the TDF-based regimen occurred in phases. In phase one, it was recommended that the TDF regimen only be given to TB/HIV co-infected patients, and pregnant and lactating women starting ART for PMTCT. In phase two, rolled out in July 2013, all HIV-infected individuals eligible for ART would receive the TDF based regimen. The findings in this study generally support the move to providing a TDF-based regimen to all HIV-infected individuals.

The results of this study should be viewed with the following limitations. The retrospective, observational nature of the study design is a limitation as data were solely based on medical records and, therefore, prone to data incompleteness. As previously reported [22,23], medical professionals may wrongly record adverse events reported by patients; this can lead to either under- or over-estimation of the event frequency. Moreover, since the ascertainment of ARV-related adverse events was solely clinical, some adverse events might have been missed, which would have led to an underestimation of the prevalence of adverse events. Despite these limitations, the study findings are useful to inform programme planning in Malawi and other comparable settings to improve care for TB/HIV patients.

## Conclusion

Implementation of the 2011 Malawi ART/PMTCT guidelines on ART initiation of TB patients was associated with increased ART uptake. However, the reduction in time between initiating TB treatment and starting ART was small, suggesting that further measures must be implemented to facilitate ART uptake. Being in HIV care prior to the start of TB treatment was associated with increased ART uptake. Increasing the early enrolment in HIV care of all HIV-positive individuals would provide greater opportunities for timely ART initiation among TB/HIV co-infected patients. The use of the TDF/3TC/EFV regimen appeared to be safer than the D4T/3TC/NVP regimen in TB/HIV patients, however, further evaluations and laboratory monitoring of ART patients on recommended TDF/3TC/EFV regimen would be beneficial in documenting adverse drug events and tolerability of the regimen.

## Competing interests

The authors declare that they have no competing interests.

## Authors’ contributions

HT, AJ conceived the study. HT, AJ, ABS, MK, WN contributed to the study design. HT drafted the manuscript and AJ, ABS, EM, WN, LG, MK, SP critically revised the manuscript. All authors gave approval of the final version to be published.

## Authors’ information

Hannock tweya is supported as an operational research fellow by the International Union Against Tuberculosis and Lung Disease, Paris, France.

## Pre-publication history

The pre-publication history for this paper can be accessed here:

http://www.biomedcentral.com/1471-2458/14/183/prepub
